# Increased incidence rates of positive blood cultures shortly after chemotherapy compared to radiotherapy among individuals treated for solid malignant tumours

**DOI:** 10.1007/s15010-022-01863-2

**Published:** 2022-06-28

**Authors:** Ashley Roen, Cynthia Terrones, Wendy Bannister, Marie Helleberg, Michael Asger Andersen, Carsten Utoft Niemann, Gedske Daugaard, Lena Specht, Amanda Mocroft, Joanne Reekie, Jens Lundgren

**Affiliations:** 1 Centre for Clinical Research, Epidemiology, Modelling and Evaluation (CREME), Institute for Global Health, University College London, London, UK; 2 PERSIMUNE Centre of Excellence, Rigshospitalet, University of Copenhagen, Blegdamsvej 9, 2100 Copenhagen ϕ, Denmark; 3 Department of Haematology, Rigshospitalet, University of Copenhagen, Copenhagen, Denmark; 4 Department of Clinical Medicine, Copenhagen University, Copenhagen, Denmark; 5 Department of Oncology, Rigshospitalet, University of Copenhagen, Blegdamsvej 9, 2100 Copenhagen ϕ, Denmark

**Keywords:** Positive blood culture, Radiotherapy, Chemotherapy, Solid malignant tumours

## Abstract

**Background:**

Cancer treatments suppress immune function and are associated with increased risk of infections, but the overall burden of serious infectious diseases in treated patients has not been clearly elucidated.

**Methods:**

All patients treated for solid malignant tumours with radiotherapy (RT) and/or standard first-line chemotherapy (C) at the Department of Oncology at Rigshospitalet between 01/1/2010 and 31/12/2016 were included. Patients were followed from treatment initiation until the first of new cancer treatment, 1 year after treatment initiation, end of follow-up or death. Incidence rates (IR) of positive blood culture (PBC) per 1000 person-years follow-up (PYFU) were calculated.

**Findings:**

12,433 individuals were included, 3582 (29%), 6349 (51%), and 2502 (20%) treated with RT, C, or both RT & C, respectively, contributing 8182 PYFU. 429 (3%) individuals experienced 502 unique episodes of PBC, incidence rate (95% CI) 52.43 (47.7, 57.6) per 1000 PYFU. The 30-day mortality rate after PBC was 24% independent of treatment modality. Adjusted incidence rate ratios in the first 3 months (95% CI) after PBC significantly varied by treatment: 2.89 (1.83, 4.55) and 2.52 (1.53, 4.14) for C and RT & C compared to RT. *Escherichia coli* (*n* = 127, 25%) was the top microorganism identified.

**Interpretation:**

PBCs are not common, but when they occur, mortality is high.

**Supplementary Information:**

The online version contains supplementary material available at 10.1007/s15010-022-01863-2.

## Introduction

Infections are a common complication among individuals diagnosed with cancer and are thought to stem from both underlying malignancy and the various treatment modalities [1]. Specifically, chemotherapy and radiotherapy can disrupt mucosal surfaces, which increases the risk of infection [2, 3]. In addition, protection from infection provided by the skin is breached with the use of medical devices and surgery. Following a malignancy, infections can arise from the dysfunctional immune system [4] and the tumour itself can also obstruct normal passages leading to abscess, fistula or empyema [5]. These disruptions can all lead to infections of the bloodstream.

However, the overall burden of infectious diseases in patients treated for cancer has not been clearly elucidated. Most research focuses on haematological malignancies [6], with few studies examining individuals treated for solid malignant tumours [7–13]. Further, the relative importance of different types of infections and infectious pathogens in patients receiving different types of cancer treatment is not well defined. A better understanding of the host factors that determine risk of infectious complications and how these factors interact with treatment-related factors could guide personalised prophylaxis, monitoring and treatment. Moreover, data on the most common infections and pathogens in cancer patients with solid tumours could be used to guide empiric treatment when patients present with fever during and after therapy.

In this study, we aimed to estimate the incidence rates of a positive blood culture (PBC) among individuals treated for solid malignant tumours with chemotherapy and/or radiotherapy. We also sought to estimate proportion who died within 30 days of the first PBC, examine the associated risk factors of 30-day mortality, and to explore the spectrum of pathogens identified among those experiencing a PBC.

## Methods

### Study population

Individuals at the Department of Oncology at Rigshospitalet, University of Copenhagen treated for solid malignant tumours with chemotherapy and/or radiotherapy between 2010 and 2016 were included in this retrospective cohort study. The aim of the study was to evaluate individuals that were on first-line chemotherapy or radiotherapy with curative intent. Therefore, individuals were excluded if they were enrolled in clinical trials, treated with non-curative intent radiotherapy, treated with very rare chemotherapy regimens (< 10 patients per regimen) or 2nd or 3rd line chemotherapy regimens or treated for relapse of malignancies.

### Data sources

Data were obtained through the Centre of Excellence for Personalized Medicine of Infectious Complications in Immune Deficiency (PERSIMUNE), a data repository of electronic health records with longitudinal nation-wide Danish data on cancer diagnoses, treatment, microbiology and pathology. Cancer diagnoses were obtained from the Danish Cancer Registry [14] and the Danish National Patient Register [15], as described previously [16]. Treatment data on radiation therapy were obtained from the ARIA® system at Rigshospitalet, University of Copenhagen, and chemotherapy data were obtained from the Danish National Patient Register, the Electronic Prescription Medication System, and Patient Protocol Administration System, as described previously [16, 17] and also included in the Supplementary Material. Briefly, the Danish Civil Registration System was used for patient demographics and the Charlson Comorbidity Index (CCI) [18] was calculated up until the first day of treatment using the coding algorithm presented by Quan et al. [19] for the updated index by Quan et al. [20] with the inclusion of cancer diagnosis. Information on blood cultures was captured from the Danish Microbiology Database [21] containing data from all clinical microbiology laboratories in Denmark from the 1st of January 2010.

### Outcome

The primary outcome was a bacterial or fungal pathogen from a blood culture, defined as a PBC during the first year following treatment initiation. For modelling purposes, the first PBC was used, but we report the spectrum of pathogens from individuals with repetitive PBCs. Within an individual, repetitive PBC with the same pathogen within 14 days of a previous episode was considered the same event, otherwise it was considered a new occurrence.

### Statistical analysis

Individuals were categorised as receiving radiotherapy (RT), chemotherapy (C), or combination (RT & C) if the two treatments were initiated within 3 months of each other. If an individual received both RT & C more than 3 months apart, they were censored at the start of the second treatment as this was likely due to a relapse or new cancer diagnosis and not part of the initial treatment plan.

Individuals treated with standard first-line therapy for solid tumours were followed up from treatment initiation on or after January 1, 2010 until 1 year after treatment initiation, a new PBC event (for modelling purposes only), 31st of December 2016, new cancer treatment initiation, death, emigration, or loss-to-follow-up, whichever came first.

Specific microorganisms from PBCs were identified and the top ten most common were tabulated.

We calculated the proportion of individuals experiencing a PBC and examined time from treatment initiation to first PBC using Kaplan–Meier methods. We calculated the incidence rates of PBC as the number of events per 1000 person-years follow-up. Since it is likely that incidence of PBC is highest shortly after treatment initiation, we stratified this analysis into three periods, namely 0–3 months, 3–6 months, and 6–12 months post-treatment initiation.

Poisson regression was used to assess risk factors associated with developing PBC, adjusting for treatment (RT, C, and RT & C), sex, age, CCI, surgery within the previous 3 months, and current cancer diagnosis. Risk factors were analysed in univariable models, and factors with *p* < 0.10 were included in multivariable models. An assumption of Poisson regression is that the incidence rate ratio (IRR) remains constant over the study period.

Among individuals with a PBC, we calculated the proportion of individuals who died within 30 days of their first PBC, stratified by treatment (RT, C, and RT & C). Using logistic regression, we examined factors associated with 30-day mortality after the first PBC. Factors investigated were the same as factors investigated for risk of PBC (RT, C, RT & C, sex, age, CCI, surgery within the previous 3 months and current cancer diagnosis) with the addition of pathogen and duration of severe neutropenia (no neutropenia, neutropenia < 7 days, neutropenia ≥ 7 days). Severe neutropenia was defined as neutrophil count less than 0.5 × 10^9^/L within 3 days of PBC, or a leukocyte count less than or equal to 2.0 × 10^9^/L if a neutrophil count was missing within three days of PBC. We followed the same model building rules as previously stated.

The study was approved by the Danish Data Protection Agency (2012‐58‐0004; RH‐2016‐47; 04433) and the Danish National Board of Health (3‐3013‐1060/1/). Analyses were performed using STATA SE 15.

## Results

### Baseline characteristics

12,433 individuals were included in the analysis, 3582 receiving RT only, 6349 receiving C only and 2502 receiving both RT & C, of which 450 received concomitant chemotherapy and radiotherapy, 772 received radiotherapy and adjuvant chemotherapy, and 1280 received neoadjuvant chemotherapy and radiotherapy. Details of the various chemotherapy compounds and the radiation schemes are given in Table 1. Overall, 53% were female, and the most common diagnosis was breast cancer [*n* = 2592 (21%)] followed by thoracic tumours [*n* = 1884 (15%)], colorectal tumours [*n* = 1384 (11%)], and head and neck tumours [*n* = 1232 (10%)]. Median age was 64 years (interquartile range, IQR: 54, 71) and CCI was 2 points (2, 3). Median follow-up time was longer among those receiving RT (365 [243, 365] days) compared to C [180 (127, 365) days] and RT & C [199 (99, 365) days] (Table 1).

**Table 1:** Baseline characteristics for individuals treated for solid malignant tumours with radiotherapy [RT], chemotherapy [C] and concomitant RT & C at Department of Oncology at Rigshospitalet, University of Copenhagen between 01/1/2010 and 31/12/2016

	Total	RT	C	RT & C
	*n* = 12,433	*n* = 3582	*n* = 6349	*n* = 2502
Total person-years follow-up	8181	2920	3746	1514
Calendar year	2013 (2011, 2014)	2012 (2011, 2014)	2013 (2011, 2015)	2013 (2011, 2015)
Female—*n* (%)	6540 (53%)	2178 (61%)	3305 (52%)	1057 (42%)
Age (median (IQR^a^))	64 (54, 71)	64 (54, 71)	64 (54, 72)	62 (54, 68)
CCI^b^	2 (2, 3)	2 (2, 3)	2 (2, 3)	2 (2, 3)
Follow-up days (median (IQR^a^))	248 (132, 365)	365 (243, 365)	180 (127, 365)	199 (99, 365)
Days on treatment (median (IQR^a^))	84 (42, 133)	36 (21, 41)	126 (120, 168)	50 (43, 125)
Surgery 0–3 months before treatment initiation—*n* (%)	8363 (67%)	2380 (66%)	4459 (70%)	1524 (61%)
*Diagnosis—n (%)*				
Breast	2592	1480 (57%)	1101 (42%)	11 (0%)
Thoracic tumours	1884	229 (12%)	1164 (62%)	491 (26%)
Colorectal	1384	85 (6%)	1137 (82%)	162 (12%)
Stomach	1231	11 (1%)	1169 (95%)	51 (4%)
Head and neck	1232	681 (55%)	18 (1%)	533 (43%)
Female genital	1149	131 (11%)	716 (62%)	302 (26%)
CNS	846	354 (42%)	70 (8%)	422 (50%)
Male genital	758	273 (36%)	431 (57%)	54 (7%)
Esophageal	420	40 (10%)	18 (4%)	362 (86%)
Bladder	303	58 (19%)	228 (75%)	17 (6%)
Other^c^	634	240 (38%)	297 (47%)	97 (15%)
*Chemotherapy*				
Platinums			4736 (75%)	1825 (73%)
Non-platinum alkylating agents			1175 (19%)	407 (16%)
Taxanes			1119 (18%)	101 (4%)
Topoisomerase inhibitors			2258 (36%)	94 (4%)
Antimetabolites			2368 (37%)	645 (26%)
Vinca alkaloids			194 (3%)	45 (2%)
Other chemotherapy^d^			578 (9%)	17 (1%)
*Radiotherapy scheme*				
< 50 Gy, < 25 days		938 (15%)		483 (8%)
< 50 Gy, 25–45 days		146 (2%)		237 (4%)
50–65 Gy, < 25 days		1 (0%)		10 (0%)
50–65 Gy, 25–45 days		1281 (21%)		964 (16%)
50–65 Gy, > 45 days		65 (1%)		48 (1%)
> 65 Gy, < 25 days		104 (2%)		27 (0%)
> 65 Gy, 25–45 days		678 (11%)		579 (10%)
> 65 Gy, > 45 days		369 (6%)		154 (3%)

Note: radiotherapy scheme < 50 Gy, > 45 days had 0 patients

^a^IQR – interquartile range

^b^Charlson Comorbidity Index

^c^Other diagnosis: neuroendocrine tumours (*n* = 206); liver and biliary tract tumours (*n* = 53); thymus cancer (*n* = 50), endocrine non-thyroid tumours (*n* = 15); soft tissue tumours (*n* = 14); skin cancer (*n* = 9); meningeal tumours (*n* = 8); kidney tumours (*n* = 4); eye tumours (*n* = 3); thyroid cancer (*n* = 1); unspecified diagnosis (*n* = 271)

^d^Other chemotherapy: cetuximab, bevacizumab, trastuzumab, bleomycin

### Pathogens

**Fig. 1:**
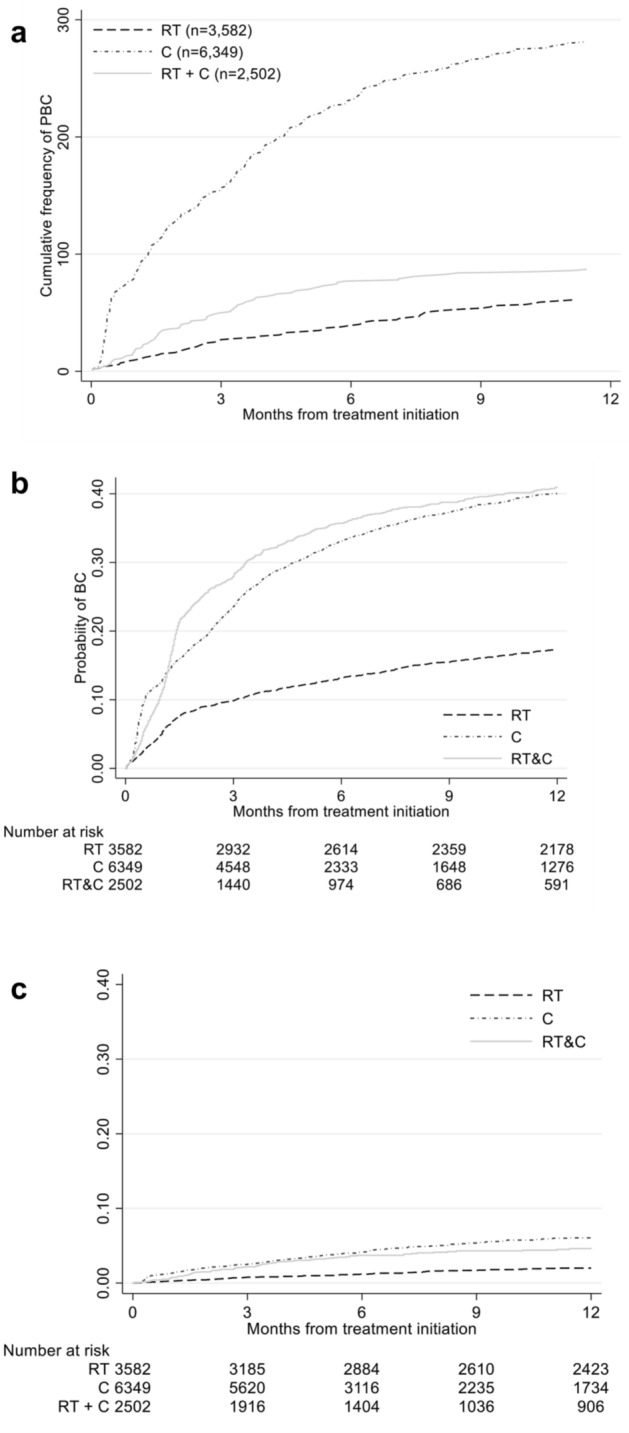
**a** Cumulative frequency of PBC within 1 year of treatment initiation. **b** Kaplan–Meier failure estimates on time from treatment initiation to first BC (blood culture) and **c** first PBC (positive blood culture) event among individuals treated for solid malignant tumours with radiotherapy (RT), chemotherapy (C) or both (RT & C) at Department of Oncology at Rigshospitalet, University of Copenhagen between 01/31/2010 and 12/31/2016

3582 (29%) of patients had at least one blood culture drawn; and there was a higher proportion of patients with drawn cultures among the C (34%), and combined RT & C (35%), than in the RT only group (16%), Chi squared *p* < 0.001. The proportion of PBC among drawn cultures was low [7%, 95% CI (6.7, 7.8)], and similar across therapy groups. There were 502 PBC episodes among 429 individuals (3%) during the follow-up period. Within the first year following treatment, 61 individuals (2%) receiving radiotherapy, 281 individuals (4%) receiving chemotherapy only, and 87 individuals (3%) receiving combination chemotherapy and radiotherapy had at least one PBC (Fig. 1). There were no differences in the proportion of PBC between the combination RT & C types, where 8 (2%), 28 (4%) and 52 (4%) had a PBC among concomitant RT & C, RT and adjuvant C, and RT and neoadjuvant C, respectively (Chi squared *p* = 0.09). RT alone tended to have fewer BC taken as well as fewer PBC compared to C and RT & C (Fig. 1). The top five pathogens were *Escherichia coli* [*n* = 127 (25%)], *Staphylococcus aureus* [*n* = 77 (15%)], *Klebsiella pneumoniae* [*n* = 55 (11%)], *Enterococcus faecium* [*n* = 31 (6%)], and *Pseudomonas aeruginosa* [*n* = 24 (5%)] (Table 2), and did not significantly vary by treatment type, *p* = 0.20. There were 18 (3.6%) cases of *Candida* spp., with similar proportions across treatment groups.

**Table 2:** Number of individuals with blood cultures drawn, the number of individuals with at least one PBC and the top ten microorganisms identified through positive blood culture stratified by treatment for individuals treated for solid malignant tumours with radiotherapy [RT], chemotherapy [C] and concomitant RT & C at Department of Oncology at Rigshospitalet, University of Copenhagen between 01/1/2010 and 31/12/2016

Result	Total	RT	C	RT + C	*p *value^*^
No. of individuals (%)	12,433 (100%)	3582 (100%)	6349 (100%)	2502 (100%)	
Number of blood cultures drawn (positive or negative)					< 0.001
0	8851 (71%)	3018 (84%)	4204 (66%)	1629 (65%)	
1	1805 (15%)	283 (8%)	1087 (17%)	435 (17%)	
2 +	1777 (14%)	281 (8%)	1058 (17%)	438 (18%)	
*Number of PBC*					< 0.001
0	12,004 (97%)	3521 (98%)	6068 (96%)	2415 (97%)	
1	373 (3%)	50 (1%)	248 (4%)	75 (3%)	
2 +	56 (< 1%)	11 (< 1%)	33 (1%)	12 (< 1%)	
No. of cases (%)	502 (100%)	74 (100%)	327 (100%)	101 (100%)	
*Top ten pathogens*					
*Escherichia coli*	127 (25%)	15 (20%)	87 (27%)	25 (25%)	0.510
*Staphylococcus aureus*	77 (15%)	13 (18%)	45 (14%)	19 (19%)	0.406
*Klebsiella pneumoniae*	55 (11%)	3 (4%)	43 (13%)	9 (9%)	0.037
*Enterococcus faecium*	31 (6%)	8 (11%)	18 (6%)	5 (5%)	0.245
*Pseudomonas aeruginosa*	24 (5%)	4 (5%)	17 (5%)	3 (3%)	0.900
*Enterococcus faecalis*	17 (3%)	2 (3%)	11 (3%)	4 (4%)	0.601
*Klebsiella oxytoca*	13 (3%)	4 (5%)	9 (3%)	0 (0%)	0.276
*Streptococcus pneumoniae*	12 (2%)	3 (4%)	6 (2%)	3 (3%)	0.074
*Candida albicans*	11 (2%)	1 (1%)	7 (2%)	3 (3%)	0.762
*Enterobacter cloacae*	11 (2%)	1 (1%)	8 (2%)	2 (2%)	0.819

^*^p Values should be interpreted with caution as performing multiple statistical tests increases the risk that significant results could be due to chance

Since there were no detectable differences in the rate of PBC or the microorganism identified between concomitant RT & C, RT and adjuvant C, and RT and neoadjuvant C, this group was collapsed into combination RT & C for the remainder of the analysis.

### Incidence rate of PBC

**Fig. 2:**
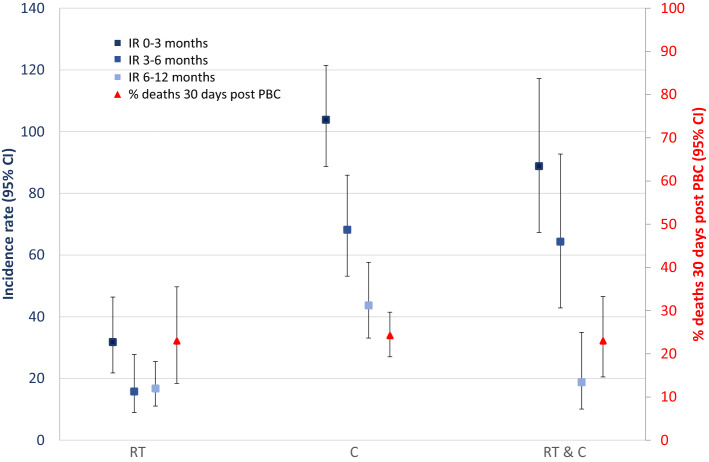
Incidence rate of positive blood culture (PBC) by time since treatment initiation (0–3 months, 0–6 months and 6–12 months) and the proportion who died within 30 days of PBC by treatment type among individuals treated for solid malignant tumours with radiotherapy (RT), chemotherapy (C) and concomitant RT & C at Department of Oncology at Rigshospitalet, University of Copenhagen between 01/1/2010 and 31/12/2016

Within the first year following treatment initiation, the overall incidence rate of PBC was 20.9 (95% confidence interval, CI 16.3, 26.8) for RT, 75.0 (66.7, 84.3) for C and 57.4 (46.5, 70.9) for RT & C per 1000 PYFU. The incidence rate of PBC was highest in the first 3 months for all treatment groups, but more than twofold higher among those receiving C (IR = 103.8 [88.7, 121.5] per 1000 PYFU) or combined RT & C [88.8 (67.3, 117.2)] compared to only RT (IR = 31.8 [21.8, 46.3]) (Fig. 2). Incidence rates of PBC decreased in the 6–12 months post-treatment [43.7 (33.1, 57.7); 18.8 (10.1, 34.9); 16.8 (11.0, 25.5); per 1000 person-years for C, RT & C, and RT groups, respectively] but remained highest in the C group.

### Predictors of PBC

In the first 3 months following treatment initiation, C and RT & C both had higher incidence rate ratios (IRR) of a PBC compared with RT alone [adjusted IRR (95% CI) 2.89 (1.83, 4.55) for C and RT; and 2.52 (1.53, 4.14) for C only]. Older age and a higher CCI score were also associated with an increased risk of PBC. Breast cancer had the lowest IRR of PBC compared to all other diagnoses. High IRR of PBC were observed in those with male genital and bladder cancers (> tenfold compared to breast cancer), which were largely based on PBC in the C group (Table 3).

**Table 3:** Multivariable predictors of positive blood culture stratified by time since treatment initiation for individuals treated for solid malignant tumours with radiotherapy [RT], chemotherapy [C] and concomitant RT & C at Department of Oncology at Rigshospitalet, University of Copenhagen between 01/1/2010 and 31/12/2016

	0–3 months post-treatment			3–6 months post-treatment			6–12 months post-treatment^a^		
	IRR	95% CI	*p *value	IRR	95% CI	*p *value	IRR	95% CI	*p *value
*Treatment*									
C versus RT	2.89	(1.83, 4.55)	< 0.001	1.71	(0.86, 3.4)	0.127	2.32	(1.4, 3.82)	0.001
RT & C versus RT	2.52	(1.53, 4.14)	< 0.001	2.72	(1.3, 5.69)	0.008	1.01	(0.48, 2.14)	0.971
*Gender*									
Females versus Males	0.997	(0.73, 1.35)	0.983	0.75	(0.48, 1.16)	0.196			
*Age*									
≥ 65 versus < 65	1.59	(1.2, 2.09)	0.001	1.76	(1.18, 2.62)	0.006			
*CCI*									
> 2 versus ≤ 2	1.58	(1.21, 2.05)	0.001	1.16	(0.8, 1.68)	0.448			
*Surgery 0–3 months prior*									
Yes versus no	0.87	(0.66, 1.15)	0.329	1.54	(1, 2.37)	0.052			
*Diagnosis*									
Breast	1			1					
Thoracic	5.02	(1.88, 13.39)	0.001	9.78	(1.21, 78.88)	0.032			
Colorectal	5.69	(2.13, 15.22)	0.001	16.07	(2.03, 127.09)	0.009			
Stomach	5.25	(1.92, 14.37)	0.001	26.81	(3.38, 212.61)	0.002			
Head and neck	7.89	(2.81, 22.18)	< 0.001	5.69	(0.63, 51.43)	0.121			
Female genital	7.31	(2.76, 19.34)	< 0.001	13.61	(1.68, 110.6)	0.015			
CNS	2.51	(0.7, 9.02)	0.158	8.99	(1.02, 79.03)	0.048			
Male genital	10.5	(3.74, 29.47)	< 0.001	6.3	(0.62, 63.47)	0.119			
Esophageal	6.03	(1.86, 19.57)	0.003	13.25	(1.47, 119.27)	0.021			
Bladder	17.17	(6.12, 48.15)	< 0.001	61.97	(7.75, 495.58)	< 0.001			
Other	23.26	(8.89, 60.83)	< 0.001	24.91	(3.05, 203.42)	0.003			

^a^Only 10 PBC events, so unadjusted model presented

Similar trends were seen 3–6 months following treatment initiation, where C and combination RT & C also had higher IRR of PBC compared to RT, older age was associated with a higher IRR of PBC, and breast cancer had the lowest IRR of PCB compared to all other diagnoses. The highest IRR of PBC observed were in those with stomach and bladder cancers, based on PBC almost exclusively in the C group (Table 3).

There were not enough PBC 6–12 months post-treatment initiation to perform a full adjusted model (*n* = 22, 50 and 10 in RT, C, and RT & C, respectively), but in a univariable analysis, IRR of PBC was significantly higher in the chemotherapy only treatment group compared to radiotherapy (Table 3).

### 30-day mortality following PBC

Overall, 24% (19.8, 28.1) (*n* = 102/429) died within 30 days of the first PBC which was similar between treatment groups [*n* = 14/61, 22.9% (13.1, 35.5); *n* = 68/281, 24.2% (19.3, 29.6); *n* = 20/87, 23.0% (14.6, 33.3) for RT, C and RT & C groups, respectively] (Fig. 2). This is higher than the 30-day mortality rate among those with a blood culture taken irrespective of the result; 15.5% (14.6, 16.3), 11.5% (9.9, 13.5), 16.5% (15.2, 17.8), and 16.6% (14.7, 18.7) for overall, RT, C and RT & C groups, respectively. In univariable models, only neutropenia lasting < 7 days was associated with 30-day mortality following PBC, OR = 1.75 (1.01, 3.04). A similar OR was observed for those with neutropenia ≥ 7 days (OR 2.01, 0.59–7.31), however, only 11 individuals had neutropenia ≥ 7 days and this association was not statistically significant (Table 4).

**Table 4:** Univariable predictors of 30-day mortality following the first positive blood culture among for individuals treated for solid malignant tumours with radiotherapy [RT], chemotherapy [C] and concomitant RT & C at Department of Oncology at Rigshospitalet, University of Copenhagen between 01/1/2010 and 31/12/2016

	No. of deaths/no. of individuals	OR	95% CI	*p *value
*Treatment*				
RT	14/61	1		
C	20/87	1.07	(0.56, 2.07)	0.836
RT & C	68/281	1.002	(0.46, 2.18)	0.996
*Neutropenic within 3 days of PBC*				
No	74/344	1		
Yes, < 7 days	24/74	1.75	(1.01, 3.04)	0.046
Yes, ≥ 7 days	4/11	2.01	(0.59, 7.31)	0.251
*Gender*				
Males	69/268	1		
Females	33/161	0.74	(0.46, 1.19)	0.217
*Age*				
< 65	39/164	1		
≥ 65 versus < 65	63/265	0.9996	(0.63, 1.58)	0.999
*CCI*				
≤ 2	50/209	1		
> 2	52/220	0.98	(0.63, 1.54)	0.944
*Surgery 0–3 months prior*				
No	45/165	1		
Yes	57/264	0.73	(0.47, 1.15)	0.179
*Diagnosis*				
Breast	1/7	1		
Thoracic tumours	19/50	3.68	(0.41, 32.95)	0.244
Colorectal	16/74	1.66	(0.19, 14.76)	0.652
Stomach	14/63	1.71	(0.19, 15.45)	0.631
Head and neck	8/31	2.09	(0.22, 20.09)	0.524
Female genital	8/40	1.5	(0.16, 14.29)	0.724
CNS	4/12	3	(0.26, 34.2)	0.376
Male genital	4/25	1.14	(0.11, 12.25)	0.912
Esophageal	3/23	0.9	(0.08, 10.33)	0.933
Bladder	7/48	1.02	(0.11, 9.85)	0.983
Other^a^	18/56	2.84	(0.32, 25.4)	0.35
*Pathogen*				
Enterobacterales	48/206	1		
Anaerobes	9/26	1.74	(0.73, 4.16)	0.211
*Candida* spp*.*	4/13	1.46	(0.43, 4.96)	0.541
*Enterococcus* spp.	10/38	1.18	(0.53, 2.59)	0.689
*Pseudomonas aeruginosa*^c^	4/20	0.82	(0.26, 2.58)	0.738
*Staphylococcus aureus*	16/64	1.1	(0.57, 2.1)	0.78
*Streptococcus* spp.	4/28	0.55	(0.18, 1.66)	0.288
Non-fermentative Gram negative	1/11	0.33	(0.04, 2.64)	0.295
*Streptococcus pneumoniae*	2/11	0.73	(0.15, 3.5)	0.696
Other^b^	4/12	1.65	(0.47, 5.7)	0.432

^a^Other diagnoses—see Table 1

^b^Other pathogen: *Campylobacter jejuni* (*n* = 2), *Listeria monocytogenes* (*n* = 2); Nocardia species (*n* = 1); *Granulicatella adiacens* (*n* = 1); *Bacillus cereus* (*n* = 1); Brevibacterium species (*n* = 1); *Rothia dentocariosa* (*n* = 1)b (*n* = 6), and *Haemophilus influenza* (*n* = 4)

^c^*Pseudomonas aeruginosa* could be classified in the other non-fermentative Gram negative, but because they represented a majority of this group, we stratified the two groups

## Discussion

Our study is the first to our knowledge to investigate the incidence rate of PBC according to treatment type in individuals with solid tumours. PBCs were not common—only 3% of our population experienced a PBC—but when they occurred, mortality was high (24%). We found significant differences in incidence rates between RT, C and combined RT & C therapies. Unsurprisingly, we also found that incidence of PBC changed over time, with the highest incidence rates observed shortly after treatment initiation (0–3 months). Those undergoing C alone or RT & C combination have a 2.9 and 2.5 fold increased risk of PBC in the first 3 months compared to those undergoing RT alone. Individuals older than 65 years at treatment initiation had a 60% increased risk of PBC in the first 3 months following treatment and breast cancer diagnosis has the lowest risk of PBC compared to all other diagnoses.

Blood culture positivity was slightly lower in our cohort (7%) compared to other studies (10–25%) [22–24]. This could be explained by variations in infection rates and practices among other countries and differences in rates of infection for haematological cancers compared to solid tumours. We acknowledge that we did not evaluate patients’ clinical status, antibiotic prescription nor hospital admission, so we could not ascertain whether there was a correlation between blood cultures drawn and a clinically significant infection. However, blood cultures are usually requested in the context of an overt infection. Individuals in our study were treated in accordance with commonly used international standards for each disease stage and cancer group, where prophylactic antibiotics are generally discouraged.

The low incidence of PBC in our cohort of patients with solid tumours was in line with a study describing patients with haematological malignancies, which found a threefold higher incidence of bacteraemia compared with oncology patients [25]. The difference between patients with haematological malignancies and patients with solid tumours was almost eight times higher in another study evaluating neutropenic patients with bacteraemia [26].

We found a higher incidence of PBC in the first 3 months after treatment. This is the period when patients are likely to be most severely immunosuppressed due to treatment, and also monitored closely and have increased contact with health services all of which may explain the higher incidence [12]. This could also be explained by survival bias as the most severely ill patients are most likely to die first. We also found highest IRRs of PBC among those with male genital and bladder cancers. Although we did not identify the source of a PBC, this could be partially explained by urinary tract infections (UTIs), as there is an established link between genitourinary cancers and UTIs [27].

Median follow-up time varied by treatment group, with those undergoing RT being followed up longer than those treated for C or RT & C combined. This could be due to our inclusion criteria, as we included only those with curative intent RT, whereas we were unable to ascertain if C was curative, palliative, or used during end-stage disease.

We identified patients with older age and a higher CCI had an increased risk of PBC. Similar results were found in a study that investigated risk factors for *E. coli* bacteraemia [28], and we have previously shown that older age is associated with neutropenic fever, where a blood was taken as a clinical suspicion of infection [16]. Tumour resections have been associated with a greater risk of developing postoperative bacteremias [29], though in our cohort, surgery was not a predisposing factor for PBC. Among 489 patients with solid tumours, the most frequent neoplasms with documented bacteraemia were hepatobiliary tumours, followed by lung cancer and lower gastrointestinal malignancies. A low proportion of patients presented with a breast cancer diagnosis, as in our cohort [11].

*E. coli* was the most common pathogen identified, followed by *S. aureus*. This corresponds with the current literature, which shows a shift from Gram-positive to Gram-negative bacteria [30]; and it contrasts with patients with haematological malignancies, where Gram-positive bacteraemias are more common [26]. Candidaemia were scarce in our cohort, as expected, since this type of infections is the most common among patients with haematological cancers which develop profound and prolonged neutropenia [31]. We did not find significant differences in pathogens by treatment group.

The overall 30-day mortality rate was consistent across treatment groups, and within the range of other studies reporting on 30-day mortality after PBC in individuals with solid neoplasms, ranging from 18 to 42% [8, 11, 13, 23, 32–34]. Of note, we only evaluated mortality in individuals with PBCs and their prognosis may be better than individuals with bloodstream infections who do not have PBC, since bacteria isolation and antibiotic resistance pattern availability could have led to adequate treatment or treatment modifications. Duration of neutropenia was the only significant factor associated with 30-day mortality; however, we were unable to fully investigate neutropenia ≥ 7 days due to few individuals experiencing this. There could also be other unmeasured factors impacting mortality that we could not investigate. CCI was not associated with 30-day mortality following a PBC in our cohort, irrespective of whether the cancer component of CCI was included or not. In contrast, a study of 773 episodes of bacteraemia among patients admitted to hospital found *E coli* was the most commonly isolated pathogen and risk of death was associated with CCI ≥ 3 [35]; and a study of 239 patients with *S. aureus* bacteremia, where a CCI ≥ 4 was associated with 30-day mortality [36], although both of these studies were not specific to individuals with cancer. Our lack of mortality risk factors identification might have been because we did not evaluate other characteristics associated with a poor prognosis in bacteraemia, such as patient clinical status, source of infection, endocarditis occurrence, pathogen resistance; nor infectious diseases consultation, which has been linked to a reduced mortality risk [37]. Results of an early intervention programme for patients with bacteraemia: analysis of prognostic factors and mortality [35].

There are some limitations that should be noted. The validity of our models depends on the untestable assumption that we appropriately adjusted for confounding. Although we adjusted for known potential confounders that were available for analysis, it is likely there are other unmeasured predictors of PBC and 30-day mortality that we could not account for. We were unable to adjust for the use of central lines or intravenous accesses which would likely be higher among those receiving C compared to RT and has a well-established connection with bloodstream infections [38].

We have shown higher rates of PBC among individuals initiating C compared to RT. This warrants further investigation; however, a comprehensive investigation into the intensity of chemotherapy and radiotherapy was beyond the scope of this manuscript. We were also unable to describe patients with radiation-induced mucositis, which may be made worse by the addition of concomitant chemotherapy and predisposes to bacterial and fungal infections [39].

We were not able to investigate administration of appropriate antibiotic treatment nor antibiotic resistance patterns, but incidence of resistance is low in Denmark [40], and would likely be evenly distributed among treatment groups. We, however, have provided a list of the most common pathogens among individuals treated for solid tumours which can provide as a basis for the spectrum of pathogens guiding early and appropriate antimicrobial therapy to individuals with solid malignant tumours diagnosed with a PBC.

PERSIMUNE is in a unique position to compare the incidence rates of PBC between different treatments due to the large amount of data. Our estimates adjust for other major factors such as surgery, CCI and diagnosis that could contribute to the incidence of PBC.

Here, we describe the microbiology aetiology of PBC among individuals treated with RT, C, or concomitant RT & C with solid tumours at a single hospital in Denmark. Although incidence of PBCs was relatively low, we found high 30-day mortality rates among individuals with a PBC. This work can provide a basis for antibiotic and antifungal treatment in patients treated for solid tumours. Further work could investigate additional risk factors of mortality among individuals with solid tumours diagnosed with PBCs, as well as the impact of previous antibiotic treatment.

## Supplementary Information

Below is the link to the electronic supplementary material.

Supplementary Figure 1: Pathogen categorizations identified among 502 unique episodes of PBC among 429 individuals and treated for solid malignant tumours with radiotherapy [RT], chemotherapy [C] and concomitant RT&C at Department of Oncology at Rigshospitalet, University of Copenhagen between 01/1//2010 to 31/12/2016. *For ease of reading, the numbers are rounded to the nearest whole number. Other includes: 3% Encapsulated bacteria (4% RT, 2% C, 6% RT+C), 3% Non-enteric Gram negative bacteria (8% RT, 2% C, 1% RT+C), 1% Other gram positive bacteria (0% RT, 1% C, 3% RT+C), 0% Other gram negative bacteria (0% RT, 1% C, 0% RT+C) (DOCX 17 KB)

Supplementary file2 (DOCX 13 KB)
