# Supplementary material for: Increased incidence rates of positive blood cultures shortly after chemotherapy compared to radiotherapy among individuals treated for solid malignant tumours
Source: Infection. 2022 Jun 28;51(1):147–57. doi: 10.1007/s15010-022-01863-2 (PMC9879832; doi:10.1007/s15010-022-01863-2)
Supplement: Supplementary file 2 — Supplementary file2 (DOCX 13 KB) [file 15010_2022_1863_MOESM2_ESM.docx]

|  | **Total** | **RT** | **C** | **RT & C** |
| --- | --- | --- | --- | --- |
| **Individuals with ≥1 PBC, n (%)** | 429 (3%) | 61 (2%) | 281 (4%) | 87 (3%) |
| 0-3 months post treatment initiation | 233 (2%) | 27 (1%) | 156 (2%) | 50 (2%) |
| 3-6 months post treatment initiation | 114 (1%) | 12 (0%) | 75 (1%) | 27 (1%) |
| 6-12 months post treatment initiation | 82 (1%) | 22 (1%) | 50 (2%) | 10 (1%) |

**Supplementary Table 1: Number of individuals with at least one positive blood culture (PBC) among those treated for solid malignant tumours with radiotherapy [RT], chemotherapy [C] and concomitant RT&C at Department of Oncology at Rigshospitalet, University of Copenhagen between 01/1//2010 to 31/12/2016**
