# Supplementary material for: Increased incidence rates of positive blood cultures shortly after chemotherapy compared to radiotherapy among individuals treated for solid malignant tumours
Source: Infection. 2022 Jun 28;51(1):147–57. doi: 10.1007/s15010-022-01863-2 (PMC9879832; doi:10.1007/s15010-022-01863-2)
Supplement: Supplementary file 2 [file 15010_2022_1863_MOESM1_ESM.docx]

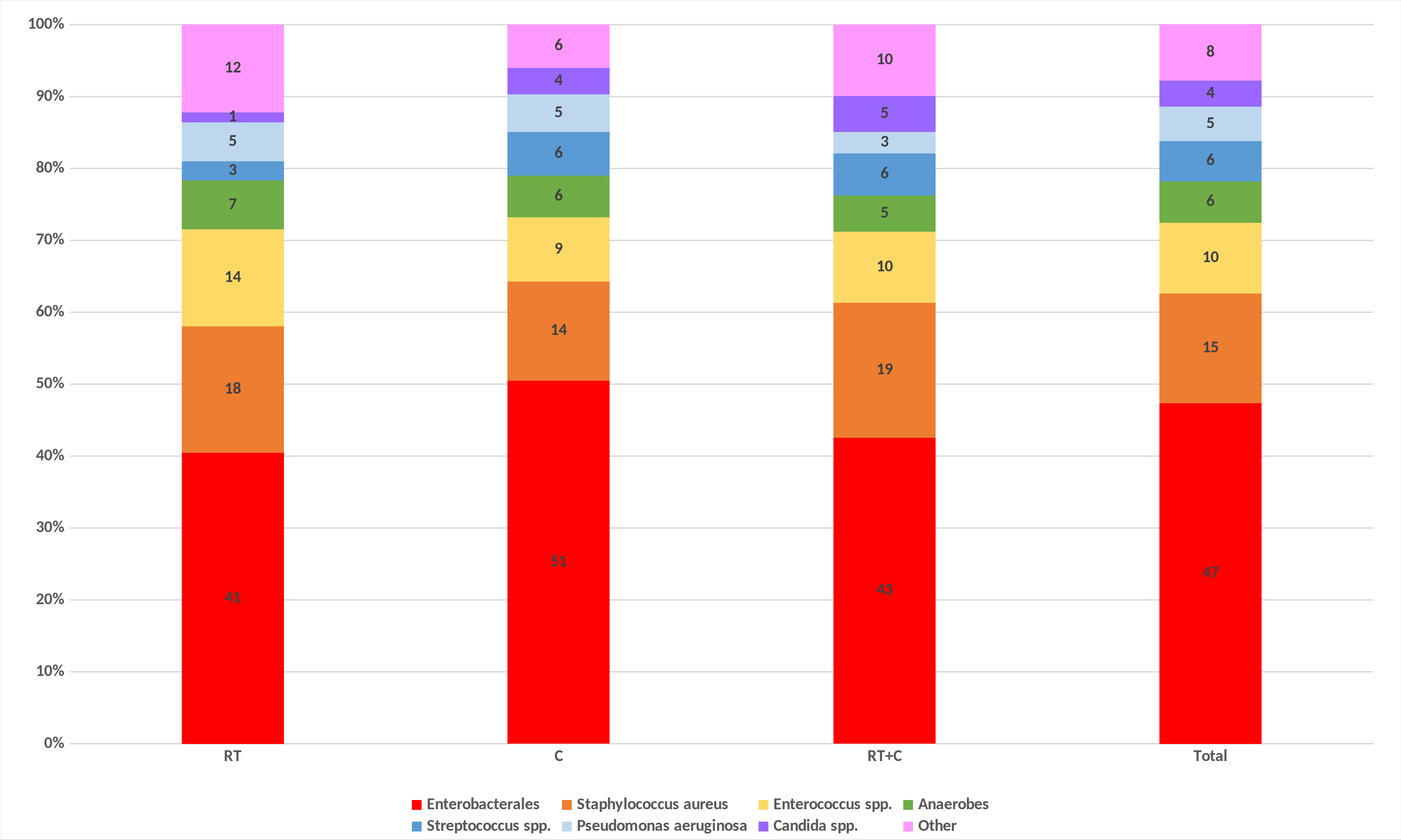
100%
90%
80%
70%
60%
50%
40%
30%
20%
10%
0%
12
1
5
3
7
14
18
41
6
4
5
6
6
9
14
51
10
5
3
6
5
10
19
43
8
4
5
6
6
10
15
47
RT
C
RT+C
Total
Enterobacterales
Staphylococcus aureus
Enterococcus spp.
Anaerobes
Streptococcus spp.
Pseudomonas aeruginosa
Candida spp.
Other
